# The implementation of frailty assessment in gynecologic oncology: an international multicenter JAGO-NOGGO survey

**DOI:** 10.1007/s00404-025-08129-w

**Published:** 2025-07-31

**Authors:** C. Flethe, C. Krause, A. von Beckerath, S. Alavi-Demirci, G. Kolb, M. Beck, K. Pietzner, J. Sehouli

**Affiliations:** 1 Young Academy of Gynecologic Oncology (JAGO), North-Eastern German Society of Gynecologic Oncology (NOGGO), Berlin, Germany; 2 Department of Gynecology, Center for Oncological Surgery, Charité-Universitätsmedizin Berlin, Corporate Member of Freie Universität Berlin, Humboldt- Universität Zu Berlin, Berlin Institute of Health, Berlin, Germany; 3 Department of Gynecology, Asklepios Klinik Hamburg Nord, Hamburg, Germany; 4 Practice for Physical and Rehabilitative Medicine and Geriatrics, Lingen, Germany

**Keywords:** Frailty assessment, Gynecologic oncology, Frailty

## Abstract

**Purpose:**

The global aging trend is expected to double the population aged 65 and older by 2050, posing new challenges for healthcare systems. Frailty is associated with poorer prognosis, increased postoperative complications, and reduced treatment tolerance. Accurate frailty assessment (FA) is therefore crucial for diagnosis, risk stratification, and individualized treatment planning. Despite its clinical relevance, clear evidence-based guidance for implementation in gynecologic oncology remains lacking**.**

**Methods:**

An anonymous online survey with 51 multiple-choice and open-ended questions was conducted from May to August 2022. It targeted gynecologists and oncologists in Germany, Austria, and Switzerland, and was distributed to 633 healthcare institutions.

**Results:**

A total of 112 responses were analyzed, revealing considerable variation in the application of frailty assessments. Only 11% reported routine use, while 36% applied FA selectively. Screening tools varied: 52% used institution-specific forms, while validated instruments such as G8 or VES-13 were rarely used. Timing was inconsistent: 49% performed FA preoperatively, 36% before chemotherapy, 31% at first presentation, and 30% without a fixed timepoint. Prehabilitation programs were largely absent; only 21% of institutions offered them. 77% of respondents indicated a need for further training.

**Conclusion:**

There are substantial gaps in the use of frailty assessments in gynecologic oncology. Standardized procedures, prehabilitation programs, and targeted education are essential to improve care quality and treatment outcomes in the context of an aging patient population.

**Supplementary Information:**

The online version contains supplementary material available at 10.1007/s00404-025-08129-w.

## What does this study add to the clinical work

**Table:** 

The findings underline the relevance of structured frailty assessment for individualized treatment planning in gynecologic oncology and point to the need for clearer implementation strategies in routine clinical care.	

## Introduction

By 2050, the global population aged 65 and older is expected to double [1]. This development poses immense challenges to healthcare systems worldwide, including gynecologic oncology, where elderly patients face heightened risks due to multimorbidity and age-related physiological changes [2, 3]. Despite these risks, elderly patients remain underrepresented in clinical trials, resulting in a lack of evidence-based strategies for this growing patient population [4]. Yet, frailty does not equate to chronological age, as it may vary significantly among individuals of the same age and thus requires independent assessment. Accurate assessment of frailty is crucial to identifying at-risk patients, developing individualized treatment plans, and optimizing therapeutic outcomes. Comprehensive Frailty Assessment (FA) plays a central role in this process. Frailty assessments consider functional, cognitive, physical, and social domains to evaluate patients’ overall vulnerability [5]. The integration of FA into clinical practice has demonstrated numerous benefits. It enables precise risk assessment and the creation of personalized treatment plans while facilitating the early identification and management of age-related health issues [6, 7]. FA ensures that patients receive appropriate treatment: frail patients are protected from overly aggressive therapies, while non-frail patients are not unjustly excluded from optimal treatments [8, 9]. A tailored approach has been shown to reduce postoperative complications and complications associated with systemic therapies, promotes faster recovery, prevents functional decline, and enhances quality of life [10–13]. FA also contributes to reducing healthcare costs, such as through shorter hospital stays and the avoidance of long-term care [10]. While the benefits of frailty assessment in gynecologic oncology are well-recognized, its routine integration into clinical practice appears limited. This study investigates the current use and integration of frailty assessments in gynecologic oncology in Germany, Austria, and Switzerland.

## Methods

This survey was initiated and conducted by the Young Academy of Gynecologic Oncology (JAGO) of the North-Eastern German Society of Gynecologic Oncology (NOGGO). It was supported by the German Society for Geriatrics (DGG). Ethical approval was obtained from the ethics committee of Charité – University Medicine of Berlin (EA1/134/21). An international online survey was developed to assess the current implementation of frailty assessment (FA) in gynecologic oncology in German-speaking countries. A questionnaire consisting of 51 multiple-choice and open-ended questions was created. While not formally validated, it was piloted by five gynecologists to ensure clarity and usability prior to distribution. It was distributed to 633 healthcare institutions, including university and non-university hospitals as well as outpatient gynecologic and oncologic practices. The survey was accessible online and conducted anonymously over four months (May to August 2022). A total of 112 responses were received, corresponding to a response rate of 18%. In line with current guidelines, we distinguished between frailty screening as an initial triage tool, and comprehensive frailty assessments as a more in-depth, multidimensional evaluation performed in patients with increased risk. The questionnaire captured various dimensions of FA implementation. It included questions on the respondents’ demographic and professional background, years of oncologic experience, and institutional affiliation. Interdisciplinary collaboration with geriatric departments was evaluated based on the presence of geriatric services. Conceptual understanding of frailty and its assessment was explored through open-ended definitions. Practical aspects of FA were assessed, such as frequency and timing (e.g., preoperative, prechemotherapy), tools used, and domains routinely evaluated. For clarity, “routine use” was defined as consistent application across all relevant patients, “occasional use” as use depending on clinical context, and “individual cases” as rare or exceptional applications. The survey further examined how FA findings influenced treatment decisions and to what extent prehabilitation concepts were known and applied in clinical practice. Educational needs in the field of FA were also addressed. Incomplete responses were included in the analysis and marked as ‘unanswered’.

The online platform SurveyMonkey.com was used for data collection, data management and data extraction. Multiple responses from the same individual were prevented by SurveyMonkey’s security features, including individualized links, cookie placement, and IP address tracking. Data analysis was performed using Microsoft Word and descriptive statistics were applied to present the results. Findings are reported as absolute numbers and percentages.

In addition to descriptive statistics, exploratory subgroup analyses were conducted using Microsoft Excel to examine potential associations between frailty screening practices and institution type, certification status, or years of clinical experience. Chi-square tests were applied, and a p-value of < 0.05 was considered statistically significant.

## Results

### Definitions of elderly patients and frailty

112 professionals from diverse clinical settings participated. Respondent characteristics are shown in Table 1.

**Table 1:** Basic Demographics of the Respondents

Characteristic	Total (N = 112)	Percentage (%)
Gender		
Female	74	66.1
Male	38	33.9
Specialization		
Gynecologists	57	50.9
Gynecologic Oncologists	46	41.1
Medical Oncologists	3	2.8
Other	6	5.4
Professional role		
Consultants	40	35.7
Specialist	27	24.1
Assistant Doctor	20	17.9
Chief Physician	16	14.3
Other	9	8
Years of experience in oncology		
< 5 years	21	18.8
5–10 years	24	21.4
10–15 years	26	23.2
> 15 years	41	36.6
Institution type		
University Hospital	36	32.1
Tertiary Care Hospital	19	17.0
Basic Care Hospital	21	18.8
Private Practice	17	15.2
Other	19	17.0
Certification status		
Certified Gynecologic Cancer Center	34	30.4
Certified Breast Center	25	22.3
Both certifications	36	32.1
No certification	16	14.3
Other	1	0.9

Percentages may not total 100 due to rounding

**Fig. 1:**
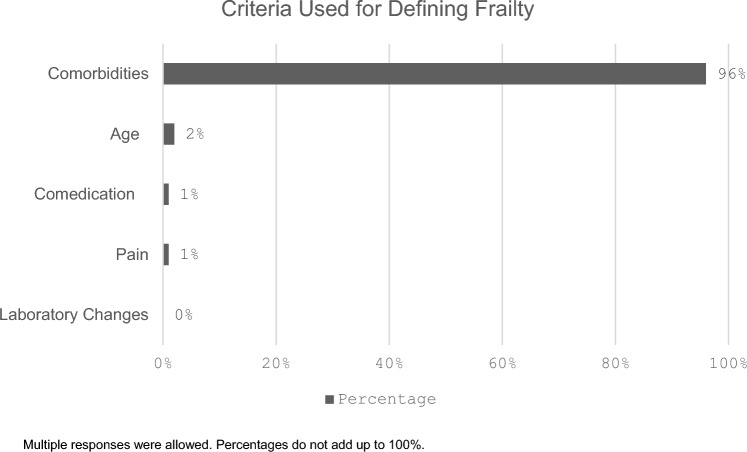
Definitions of Frailty Among Respondents. Multiple responses were allowed. Percentages do not add up to 100%

Among the participants, there was no clear consensus on the definition of an elderly patient, as perspectives varied widely among respondents: 30% (n = 32/107) considered patients aged ≥ 70 years as elderly, 25% (n = 27/107) ≥ 75 years, 12% (n = 13/107) ≥ 80 years, 8% (n = 9/107) ≥ 65 years, and 2% (n = 2/107) ≥ 60 years. 23% (n = 25/107) stated that an elderly patient could not be clearly defined. The survey indicated that frailty is predominantly defined by comorbidities (96%, n = 102/106), while only a small proportion attributed it to age (2%, n = 2/106) or to factors like comedication and pain (1%, n = 1/107). Laboratory changes were not cited as relevant (Fig. 1).

### Therapy adjustments

96% (n = 103/107) of participants reported therapy adjustments due to comorbidities. Although frailty was defined by age in only 2% (n = 2/107) of cases, 12% (n = 13/107) of therapy modifications were nevertheless based on the patient’s age. 2% (n = 2/107) reported therapy changes due to pain, and 5% (n = 5/107) due to comedication. No adjustments were reported based on albumin levels.

### Frailty screening

Only 11% (n = 12/107) of participants regularly performed frailty screening, while 36% (n = 39/107) did in individual cases, and 50% (n = 54/107) did not perform it at all.

Exploratory analyses examined differences in frailty screening by institution type, certification, and experience. No significant associations were found (institution: χ^2^ = 6.52, p = 0.59; certification: χ^2^ = 2.43, p = 0.30; experience: χ^2^ = 9.02, p = 0.17), though trends showed higher usage in university hospitals, certified centers, and among more experienced clinicians.

**Fig. 2:**
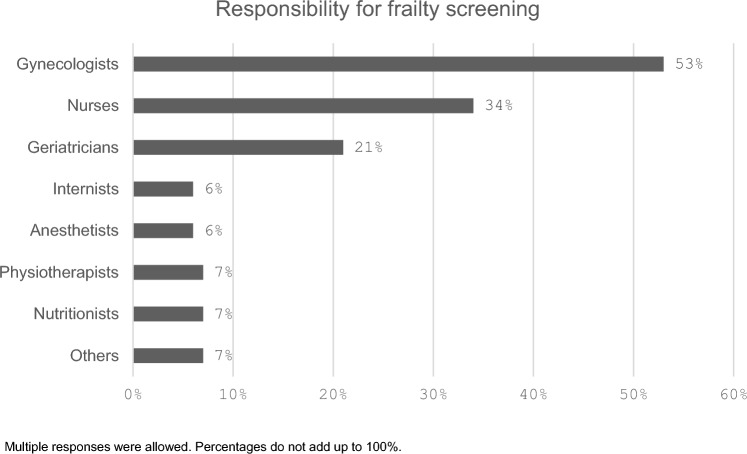
Screening Responsibility. Multiple responses were allowed. Percentages do not add up to 100%

The responsibility for screening varied, with 53% (n = 30/53) done by gynecologists, 34% (n = 19/56) by nurses, 21% (n = 12/56) by geriatricians, 7% (n = 4/56) by physiotherapists and nutritionists and 6% (n = 9/56) by anesthetists or internists (as shown in Fig. 2).

Frailty evaluation occurred at different stages: 49% (n = 27/55) preoperatively, 36% (n = 20/55) before starting chemotherapy, 31% (n = 17/55) at the first presentation, and 30% (n = 16/55) without a fixed time. Additional diagnostic measures were taken in 76% (n = 50/66) of individual cases before starting therapy for frail patients.

### Instruments used for frailty screening

Most respondents (52%, n = 27/52) used an individual clinic assessment form for frailty evaluation. Other tools included self-reported instruments such as the Vulnerable Elders Survey-13 (VES-13) and the G8 Screening Questionnaire, each used by 10% (n = 5/52) of respondents, and the Groningen Frailty Index (GFI), used by 8% (n = 4/52). Additional tools like the Triage Risk Screening Tool (TRST) were employed by 6% (n = 3/52), while the Fried Frailty Criteria and the Abbreviated Comprehensive Geriatric Assessment (aCGA) were each used by 4% (n = 2/52). Furthermore, 17% (n = 9/52) reported using other unspecified instruments.

### Contents of frailty assessments

During medical history-taking, participants routinely assessed patients’ living situation and social background (89%, n = 90/101) as well as their functional status and ability to perform activities of daily living (79%, n = 80/101). Tools used include individual clinic forms (47%, n = 47/101), the Barthel Index (38%, n = 38/101), and other various tools (15%, n = 15/101). Most respondents (92%, n = 93/101) documented all comorbidities, using individual listings (66%, n = 67/101), clinic-specific forms (38%, n = 38/101), and the Charlson Comorbidity Index (7%, n = 7/101).

### Medication management

Home medication was documented by 98% (n = 99/101), primarily by doctors (93%, n = 94/101), nurses (32%, n = 32/101), and others (3%, n = 3/101). Before surgery, 31% (n = 31/100) always, 52% (n = 52/100) occasionally, and 17% (n = 17/100) never reviewed medication. Similar patterns were reported before chemotherapy (34% always, 52% occasionally, 14% never). Drug interactions were checked by 50% (n = 48/96), 32% (n = 31/96) in individual cases, and 18% (n = 17/96) not at all—mainly by gynecologists (64%, n = 55/86), internists (15%, n = 13/86), or anesthesiologists (2%, n = 2/86), using software (47%, n = 41/87) or institutional pharmacies (43%, n = 37/87).

### Performance and functional status assessment

For assessing patients' general condition, 89% (n = 88/99) reported a uniform approach at their clinics, primarily using the ECOG (79%, n = 18/99) and the Karnofsky Index (72%, n = 71/99). The ASA classification was used by 46% (n = 46/99), while 7% (n = 7/99) used individual assessment forms or personal estimates.

### Falls risk assessment

Falls risk was routinely assessed during anamnesis by 38% (n = 38/101) of participants, occasionally by 24% (n = 24/101), and not assessed at all by 37% (n = 37/101). Tools included clinic-specific forms (46%, n = 30/65), the Esslinger Fall Risk Assessment (37%, n = 24/65), the Timed-Up-and-Go test (22%, n = 14/65), and hand strength tests (14%, n = 9/65).

### Nutritional status assessment

Nutritional status assessment was performed by 67% (n = 68/101) of participants during anamnesis, 30% (n = 30/101) in individual cases, and 3% (n = 3/101) did not assess at all. Common tools include BMI and body weight (66%, n = 65/98), weight loss (44%, n = 23/98), albumin level (25%, n = 24/98), BIA measurement (13%, n = 13/98), and clinic-specific forms (16%, n = 16/98).

### Cognitive and psychological domains

Mental status was assessed in individual cases by 45% (n = 45/100), always by 32% (n = 32/100), and not at all by 23% (n = 23/100). Tools included individual forms 56% (n = 38/68) and the Mini-Mental Test (29%, n = 20/68), with other tools used in 13% (n = 13/101) of cases.

Fatigue and depression were not assessed by 44% (n = 44/101), assessed case-by-case by 39% (n = 39/101), and consistently assessed by 13% (n = 13/101), and 5% (n = 5/101) of participants were uncertain whether fatigue and depression were assessed in their respective departments. Assessment methods included individual forms (65%, n = 33/51), the Hospital Anxiety and Depression Scale (HADS) (20%, n = 10/51), the Brief fatigue Inventory (8%, n = 4/51), and other tools (18%, n = 9/51).

### Other health aspects

**Fig. 3:**
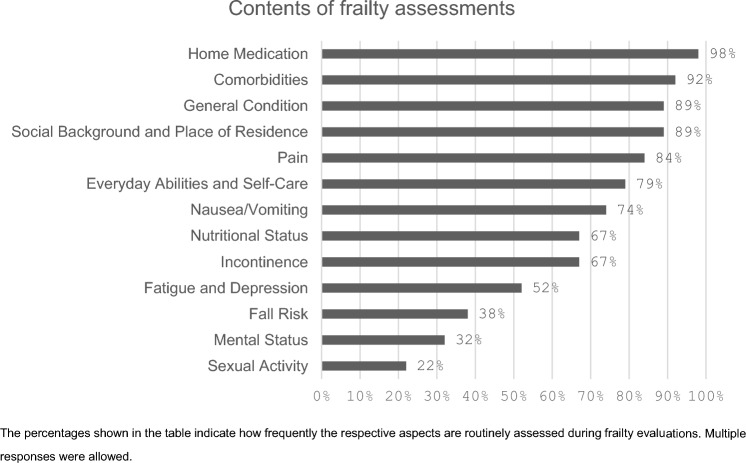
Contents of frailty assessments. The percentages shown in the table indicate how frequently the respective aspects are routinely assessed during frailty evaluations. Multiple responses were allowed

Regularly recorded aspects in medical history included nausea/vomiting (74%, n = 73/99), incontinence (67%, n = 66/99), pain (84%, n = 83/99), and sexual activity (22%, n = 22/99), though 12% (n = 12/99) did not record these aspects at all (as shown in Fig. 3).

### Frailty and prehabilitation programs

Programs to address frailty were notably lacking, with 65% (n = 72/110) of respondents either unaware of or without access to such programs. Similarly, at least 65% (n = 72/110) lacked a prehabilitation concept before surgery or chemotherapy, while 21% (n = 24/110) confirmed its presence, and 14% (n = 14/110) were uncertain.

### Educational needs

In terms of frailty assessment education, 77% (n = 84/109) of the participants expressed a desire for further training, while 23% (n = 25/109) reported no such need.

## Discussion

We are not aware of any previous studies that have systematically assessed the implementation of frailty assessments in gynecologic oncology across Germany, Austria, and Switzerland. The findings reveal significant gaps in integrating FAs into routine clinical practice and highlight the urgent need for a standardized, comprehensive approach to improve the care of gynecologic oncology patients.

Defining who is considered as “elderly” remains inconsistent in clinical and research contexts. While age is often used as a simple cutoff, it does not necessarily reflect a person’s overall health or functional status. In our survey, 2% (n = 2/107) of respondents considered people aged 60 to be elderly, and 8% (n = 9/107) used a threshold of 65 years. Most chose a higher cutoff: 30% (n = 32/107) defined individuals over 70 as elderly, and 25% (n = 27/107) used 75 years. 23% (n = 25/107) stated that an elderly patient could not be clearly defined. This variation reflects the lack of a clear definition in the scientific literature. Because chronological age alone does not account for individual differences in health and resilience, there is growing interest in assessing frailty - a clinical state of increased vulnerability to stressors and adverse outcomes. Unlike age, frailty is dynamic and can occur at different points in life [11]. Our findings indicate that respondents primarily associated frailty with the presence of comorbidities, with 96% (n = 103/107) identifying them as a key factor in their definition. Notably, 12% (n = 13/107) of participants reported adjusting therapy based solely on chronological age, even though only 2% (n = 2/106) explicitly linked frailty to age. In contrast, relevant factors such as polypharmacy and pain were largely overlooked, each cited by only 1% (n = 1/107). This suggests a narrow interpretation of frailty that may miss key aspects of overall health. Incorporating frailty into clinical decisions enables a more personalized approach, guided by functional status, comorbidities, and resilience rather than age alone [12], supporting the principles of individualized care. In our survey, only 11% (n = 12/107) of respondents routinely performed frailty screenings, underscoring challenges in the structured identification of vulnerable patients. As comprehensive assessments typically follow a positive screening, limited screening use also implies low overall implementation. Screening methods were largely non-standardized: 52% (n = 27/52) used clinic-specific forms, while only 10% (n = 5/52) applied the validated such as the G8. This lack of standardization may lead to inconsistent or delayed identification of at-risk patients. The German S3 guidelines recommend a two-step approach: initial screening with the G8 for patients aged ≥ 70, followed by comprehensive assessment for those scoring ≤ 14 [14]. This strategy enables more targeted care and efficient resource use. Following a positive screening result, a comprehensive frailty assessment should cover multiple clinically relevant domains, including functional status, comorbidities, cognitive function, nutrition, psychological health, social support, and polypharmacy [5]. While some domains were well-represented in clinical practice - 79% (n = 80/101) of respondents assessed functional status, 92% (n = 93/101) documented comorbidities, and 89% (n = 90/101) evaluated social support - others were frequently overlooked. Notably, cognitive function was routinely assessed by only 32% (n = 32/101), and nutritional status by 67% (n = 68/101), with few respondents utilizing objective measures such as serum albumin levels. Fatigue and depression, essential components of psychological health, were assessed by just 13% (n = 13/101). These findings suggest that psychological health remains an underrepresented component in current frailty assessment practices. The timing of FAs varied across institutions. Preoperative assessments (49%, n = 27/55) and evaluations before initiating chemotherapy (36%, n = 20/55) were the most common, while 31% (n = 17/55) of respondents conducted FAs during the initial patient presentation. The German S3 guideline on comprehensive geriatric assessment recommends at least a preoperative or pretherapeutic screening for at-risk groups [14]. Given the dynamic nature of health status in oncologic patients, regular reassessments may be necessary to ensure that therapeutic strategies remain aligned with the patient’s evolving condition.

Despite growing evidence supporting the use of frailty assessments in oncology, their implementation in daily clinical practice remains limited. One of the most frequently reported barriers is the perception that frailty assessments are too time-consuming or offer limited added value. A survey by Dale et al. found that these concerns are often exacerbated by high patient volumes and resource constraints in busy clinical settings, making it difficult for healthcare providers to prioritize FAs [15]. Another challenge is the fragmented responsibility for conducting FAs. In our study, gynecologists performed the majority of assessments (53%, n = 30/56), followed by nurses (34%, n = 19/56) and geriatricians (21%, n = 12/56). The lack of clear role assignments and specified workflows limits the effective integration of FAs into routine care. Addressing this issue requires stronger interdisciplinary collaboration, ensuring that oncologists, geriatricians, nurses, and allied health professionals work together to deliver FAs consistently and comprehensively. Additionally, the lack of protocols further impedes FA implementation. While tools such as ASCO’s Practical Geriatric Assessment provide frameworks [16], they are not widely adopted. Another issue is the limited awareness and knowledge of FA guidelines. A large-scale ASCO survey involving 1,277 oncology providers found that only 52% were aware of the 2018 FA recommendations. Even among those familiar with the guidelines, knowledge gaps and insufficient training were frequently reported [15]. Incorporating frailty assessments into digital health systems may offer opportunities to improve efficiency and consistency in clinical routines.

Prehabilitation programs, designed to improve physical and psychological resilience before treatment, remain underdeveloped [17]. In our study, at least 65% (n = 72/110) of respondents reported the absence of such programs in their institutions. Prehabilitation combining physical training, nutrition, and psychosocial care has the potential to improve clinical outcomes in frail patients [18]. Developing and implementing these programs should be a priority, particularly for frail patients undergoing intensive oncologic therapies.

This study has several limitations: The response rate of 18%, although within the expected range, may introduce response bias, as clinicians with an interest in frailty assessments may have been more likely to respond. The questionnaire was piloted but not formally validated. Moreover, data were self-reported and may be subject to recall or social desirability bias.

## Conclusion

This survey shows that frailty assessments are still rarely used in routine care and that prehabilitation programs are frequently lacking. The results indicate a need for more training opportunities and suggest that improved interdisciplinary collaboration and stronger institutional support could facilitate broader implementation. Systematic integration of frailty assessments into clinical workflows may support more individualized treatment decisions in gynecologic oncology patients.

## Supplementary Information

Below is the link to the electronic supplementary material.

Supplementary file1 (DOCX 20 KB)

## Data Availability

The dataset generated and analyzed during this survey is not publicly available due to participant confidentiality and institutional policy but may be obtained from the corresponding author upon reasonable request.
