# Supplementary material for: The implementation of frailty assessment in gynecologic oncology: an international multicenter JAGO-NOGGO survey
Source: Arch Gynecol Obstet. 2025 Jul 31;312(4):1337–44. doi: 10.1007/s00404-025-08129-w (PMC12414013; doi:10.1007/s00404-025-08129-w)
Supplement: Supplementary file 1 — Supplementary file1 (DOCX 20 KB) [file 404_2025_8129_MOESM1_ESM.docx]

**Supplementary Material 1: Survey Questionnaire (English Translation)**

**F1. Age**
Free text

**F2. Gender**

- Male
- Female

**F3. Postal Code**
Free text

**F4. What is your position in the clinic?**

- Resident
- Specialist
- Senior physician
- Head of department
- Other (please specify)

**F5. How many years of experience do you have treating cancer patients?**

- < 5 years
- 5–10 years
- 10–15 years
- 15 years

**F6. Function**

- Gynecologist
- Gynecologic oncologist
- Oncologist
- Other (please specify)

**F7. Your workplace is:**

- Private practice
- Primary care hospital
- Secondary care hospital
- Tertiary care hospital
- University hospital

**F8. Number of gynecologic-oncologic surgeries per year (excluding minor procedures):**

- None
- < 30
- 30–50
- 50–100
- 100
- I don’t know

**F9. Number of gynecologic patients receiving chemotherapy per year (including outpatient clinics):**

- None
- ≤ 50
- 50–100
- 100–200
- 200–500
- 500
- I don’t know

**F10. Certification status of your clinic:**

- Certified gynecologic cancer center
- Certified breast center
- Both
- No certification
- Other certification

**F11. How do you educate yourself on geriatric oncology? (Multiple answers possible)**

- Congresses
- Publications
- Seminars
- Internet
- Not at all
- Other (please specify)

**F12. Would you like more training opportunities on geriatric assessment?**

- Yes
- No

**F13. Do you conduct (gyneco-)oncologic studies?**

- Yes
- No

**F14. If yes: reasons for patients not participating (Multiple answers):**

- Not meeting inclusion criteria
- Patient refusal
- Limited communication
- Age-related exclusion by investigator

**F15. Is there a prehabilitation concept in your clinic?**

- Yes
- No
- Don’t know

**F16. What age defines an “elderly patient” in your opinion?**

- 60 years
- 65 years
- 70 years
- 75 years
- 80 years
- I cannot define clearly

**F17. Is general condition (ECOG, ASA, Karnofsky) routinely discussed in tumor boards?**

- Always
- Sometimes
- No
- I don’t know

**F18. What is the most important factor for frailty in your opinion?**

- Age
- Comorbidities
- Polypharmacy
- Lab changes
- Pain

**F19. Based on which factor is therapy adjusted most often?**

- Age
- Comorbidities
- Polypharmacy
- Albumin
- Pain
- Other

**F20. Is there a geriatric department or geriatrician in your hospital?**

- Yes
- No
- I don’t know

**F21. If yes: is there structured collaboration?**

- Yes
- No
- I don’t know

**F22. Is frailty screening performed?**

- Routinely
- In selected cases
- Not at all
- I don’t know

**F23. If yes: who performs it? (Multiple answers)**

- Gynecologist
- Geriatrician
- Anesthesiologist
- Internist
- Physiotherapist
- Nurse
- Nutritionist
- Other

**F24. What screening tools are used? (Multiple answers)**

- VES-13
- G8
- TRST
- Fried Frailty Criteria
- Abbreviated CGA
- GFI
- Institutional form
- Other

**F25. When is frailty assessed? (Multiple answers)**

- No fixed time
- First consultation
- Preoperative
- Postoperative
- Before chemotherapy
- During chemotherapy
- After chemotherapy
- Regularly (please specify interval)

**F26. If screening is positive, are further diagnostics performed?**

- Always
- Sometimes
- No

**F27. Are there structured programs to improve frailty?**

- Yes
- No
- Don’t know

**F28. Is the social background assessed? (Multiple answers)**

- Living situation
- Social support
- Not assessed
- Other

**F29. Are activities of daily living documented?**

- Yes
- No

**F30. If yes: in what percentage of cases (estimate)?**

- 25%
- 50%
- 75%
- 100%

**F31. If yes: which tools are used? (Multiple answers)**

- Barthel Index
- IADL
- PGBA
- Lachs Screening
- Institutional form
- Other

**F32. Are comorbidities documented?**

- Always
- Sometimes
- No

**F33. If yes: how are they documented? (Multiple answers)**

- Charlson Index
- CIRS-G
- Institutional form
- Individual listing
- Other

**F34. Is home medication documented?**

- Always
- Sometimes
- No

**F35. If yes: who documents it? (Multiple answers)**

- Physician
- Nurse
- Other

**F36. Is home medication optimized before surgery?**

- Always
- Sometimes
- No

**F37. Is home medication optimized before chemotherapy?**

- Always
- Sometimes
- No

**F38. Are drug interactions checked before chemotherapy?**

- Always
- Sometimes
- No

**F39. If yes: who checks interactions? (Multiple answers)**

- Gynecologist
- Geriatrician
- Anesthesiologist
- Internist
- Other

**F40. If yes: how are interactions checked? (Multiple answers)**

- Software/Internet
- Hospital pharmacy
- Other

**F41. Is a uniform measure of general condition used in your clinic?**

- Yes
- No

**F42. If yes: which tool? (Multiple answers)**

- ECOG
- Karnofsky
- ASA
- Institutional form
- Clinical judgment
- Other

**F43. Is fall risk assessed?**

- Always
- Sometimes
- No

**F44. If yes: which tools? (Multiple answers)**

- Grip strength
- Fall risk scale (e.g., Esslingen)
- Timed Up and Go
- Tandem stand
- Institutional form
- Other

**F45. Is nutritional status assessed?**

- Always
- Sometimes
- No

**F46. If yes: which tools? (Multiple answers)**

- Body weight
- BIA
- Weight loss
- BMI
- Albumin
- Institutional form
- Other

**F47. Is mental status assessed?**

- Always
- Sometimes
- No

**F48. If yes: which tools? (Multiple answers)**

- MMSE
- SKT
- DemTect
- TFDD
- Institutional form
- Other

**F49. Is fatigue and depression assessed?**

- Always
- Sometimes
- No
- I don’t know

**F50. If yes: which tools? (Multiple answers)**

- Brief Fatigue Inventory
- GDS (30-item)
- HADS
- Institutional form
- Other

**F51. Are the following routinely recorded? (Multiple answers)**

- Incontinence
- Nausea/vomiting
- Pain
- Sexual activity
- None of the above
